# Determinants of Ruminant Farmers' Use of Sustainable Production Practices for Climate Change Adaptation and Mitigation in Enugu State, Nigeria

**DOI:** 10.3389/fvets.2022.735139

**Published:** 2022-02-07

**Authors:** Cynthia Ebere Nwobodo, Blessing Nwokolo, Juliana Chinasa Iwuchukwu, Violet Amarachukwu Ohagwu, Remigius Ikechukwu Ozioko

**Affiliations:** Department of Agricultural Extension, University of Nigeria, Nsukka, Nigeria

**Keywords:** animal welfare, ruminant production, climate change, sustainable agriculture, adaptation and mitigation

## Abstract

A sustainable ruminant production system ensures economically viable livestock systems that meet the current and future demands of animal products as well as the environmental safety of current and future generations. The study analyzed the determinants of ruminant farmers' use of sustainable production practices for climate change adaptation and mitigation in Enugu State, Nigeria. Multistage sampling procedure was used to select ninety six (96) ruminant farmers that constituted the sample for the study. Semi-structured interview schedule with open ended questions was used in data collection. Data were analyzed using multiple regression and Pearson Moment Correlation statistics. Access to veterinary services (*t* = 2.056, *p* = 0.044), monthly household income (*t* = 3.582, *p* = 0.001) and annual income from ruminant production (*t* = −2.635, *p* = 0.011) were socio-economic factors that significantly influenced use of sustainable practices. The adjusted R- square implies that the three factors were able to explain 24% of variance in use of sustainable practices. There is a significant positive correlation (*r* = 0.426, *p* = 0.000) between knowledge level of farmers and their use of sustainable production practices. Schemes for financial inclusion such as payment for ecosystem services can spur farmers to adopt mitigation strategies. Improved climate change knowledge can enhance ruminant farmer's resilience to the increasing impacts of climate change.

## Introduction

Since the Rio meeting of 1992, the world has committed to reducing greenhouse gases which cause climate change and to invest in processes that reduce the impact of climate change on lives and livelihoods of populations. Climate change adaptation and mitigation have therefore been on the front burner of scientific research, attracting international debates, and consensus. The emergent of numerous climate change research gave rise to interests in sustainability and with the Sustainable Development Goals coming on board, researchers and development practitioners have directed efforts to sustainable production. Ruminant production and climate change are strongly inter-dependent. The ruminant sector contributes to greenhouse gases (GHGs) emissions mainly through the emission of methane (CH_4_), largely from enteric fermentation, nitrous oxide (N_2_O) emission from manure and the use of nitrogenous fertilizers in growing feed, and carbondioxide (CO_2_) from fossil fuel burning (1). Globally, ruminants contribute about 80 percent of livestock emissions of carbon dioxide (CO_2_), 47 percent of Methane (CH_4_), and 24 percent of Nitrous oxide (N_2_O) emissions (2, 3). About 90% of livestock emissions are produced by ruminants alone through enteric fermentation (4). Enteric fermentation methane (CH_4_), emitted from ruminants during digestion, is a main source of global methane emissions and is responsible for 25% of global methane emissions or 4% of overall anthropogenic GHG emissions (5).

As ruminant production increases worldwide due to increasing population, global increase in income and consequent increase in demand for animal protein, wastes from ruminant production is becoming a serious environmental concern. The expansion in production of ruminant animals in response to the increasing demand leads to resultant expanded quantities and areas of production. These have detrimental effects on the environment (6, 7). Obviously, in spite of their growing global importance, livestock are increasingly being held responsible for many adverse effects on the environments including climate change, loss of vegetation cover, reduced biodiversity, soil erosion and compaction, and excessive run-off often from overgrazing (8).

On the other hand, ruminants are adversely affected by the detrimental effects of extreme weather events. Climate change extremes and seasonal fluctuations in herbage quantity and quality affects the well-being of livestock leading to declines in production and reproduction efficiency (9). For instance, temperature affects most of the critical factors for ruminant production, such as water and feed availability, production, reproduction and health of animals (10). High temperatures predisposes ruminants to physiological stress and diseases. Also, high temperatures trigger the incidence of transmittable chronic respiratory diseases: *coryza*, salmonellosis and infectious *laryngotracheritis* (11). Thermal livestock stress decreases feed intake and efficiency of feed conversion, especially for livestock that are fed large amounts of high-quality feeds. In the case of cattle, feed intake reduction leads to a negative energy balance and reduced weight gain (10). Climate change affects feed availability and quality leading to nutrient deficiencies resulting in metabolic disease of varying nature. Mineral deficiencies results in anemia, retarded growth, and reproductive disorders in livestock. Also, the nutritional stress increases the case of pregnancy toxemia and neonatal death due to poor milk yield and resultant reduction in immunity with consequent proneness to many infectious diseases (12).

Vector-borne diseases are also highly influenced by climatic factors. Climate change result to an increased spread of existing vector-borne diseases and macro-parasites of animals as well as the emergence and spread of new diseases (13). According to Ashraf et al. (14), climate change exerts both direct and indirect influences on the transmission of vector borne diseases, affecting timing of outbreak or the intensity of an outbreak, establishing a temporal linkage and affecting geographical distribution, establishing a spatial linkage of many infectious diseases in animals. Prolonging of the warm season due to climate change may increase the number of cycles of infection possible within 1 year for warm- or cold-associated diseases, respectively. As climate change disrupts rainfall patterns, there are high risks of a number of infectious diseases of ruminants including zoonotic illnesses. For instance, under high humidity, the incidence of *helmenthosis* increases in ruminants (11). Also, prolonged period of no rainfall leads to drought spells which affects pasture availability, quantity and quality. Feed scarcity due to limited pasture leads to stress, immunosuppression and finally predispose ruminant animals to different diseases and death.

Ruminant production is very important to Nigeria's economy not only as source of animal protein but as source of livelihood for the rural poor farmers. In Southeast Nigeria, ruminants are specially raised as source of investment in which they could serve as source of income for household expenses, meat, and manure, used in social and religious ceremonies and as a source of insurance against crop failure (15) especially resulting from climate-related shocks. Sheep and goat accounts for majority of the ruminant production in Enugu State with the cattle being produced mainly for ceremonial activities. All members of the household including men, women and children are involved in the management of ruminant production in the area. Enugu State, ruminant production is faced with numerous challenges ranging from seasonal feed shortages, high mortality rate as a result of diseases and poor access to veterinary services, low reproduction and general sub-optimal management practices. Climate change further increases the already overwhelming risks facing ruminant production in the area.

The strong inter-dependence between ruminant animal production and climate change calls for concerted efforts toward sustainable production of ruminant animals. Hoving et al. (16) noted that sustainable intensification is critical to global production of animal protein and for the farmers and livelihoods that are dependent on livestock. Sustainable agriculture is an agriculture that must produce adequate amounts of high-quality food, protect its resources and be both environmentally safe and profitable (17). Sustainability rests on the principle that the needs of present generation should be met without compromising the ability of future generations to meet their own needs (18). Sustainable production practices are those practices that in as much as they increase production, does little or no harm to the environment. Therefore, the quest to increase ruminant production to meet growing demand should not be at the detriment of the environment. Ensuring economically viable livestock systems that meet the current and future demands of animal products as well as the environmental safety of current and future generations are the interests of a sustainable ruminant production system. Sustainable intensification of ruminant production can be achieved by improving animal health, welfare and production, without harming the environment (19). Hence, adaptation and mitigation strategies in ruminant production need to recognize the unique challenge to decrease absolute emissions, largely through reduced emissions intensity, while meeting the growing global demand for meat and animal products (20). The FAO (2) noted that improving feeding practices and digestibility of diets, improving yields through genetics, feeding practices and animal health, reducing land use change from feed crop cultivation and pasture expansion, improving manure management, and improving the efficiency of feed crop production, are potential mitigation pathways in ruminant production. Hence, the following questions suffice: Do ruminant farmers use sustainable production practices? Therefore, the study sought to: identify sustainable production practices of ruminant farmers for climate change adaptation and mitigation, determine socio-economic factors influencing farmers' use of sustainable practices, determine the relationship between ruminant farmers' knowledge level and their use of sustainable production practices and examine the challenges encountered by ruminant farmers in using sustainable production practices.

## Theoretical Framework

The Theory of Planned Behavior (TPB) has been largely applied to the study of environmental science research as it can provide valuable implication not only in predicting and managing individual behavior, but also for increasing social and environmental sustainability (21). This paper is based on TPB) which stems from the Theory of Reasoned Action (TRA). The TRA posits that attitude and subjective norms are the determinants of intention, and that intention directly affects behavior to some extent (22). In the TPB, individual intention mainly depends on three determinants: attitude, subjective norms, and perceived behavioral control (21). The theory posits that behavioral intentions are influenced by the attitude about the likelihood that the behavior will have the expected outcome and the subjective evaluation of the risks and benefits of that outcome (23). The two theories are based on the premise that individuals make logical, reasoned decisions to engage in specific behaviors by evaluating the information available to them (24). The performance of a behavior is determined by the individual's intention to engage in it, which is influenced by the value the individual places on the behavior, the ease with which it can be done and the views of significant others and the perception that the behavior is within his/her control. This means that individuals will adopt a behavior which they think they can benefit from and which they have capacity to use within their own specific circumstances and which its adoption is supported by the members of their social system. Knowledge is a prerequisite for effective action (25). Li et al. (26) noted that factors which influence pro-environmental behavior of individuals include environmental knowledge, demographic factors, institutional factors, economic factors, social and cultural factors, motivation, and so on. Farmers' knowledge about a technology is often influenced by their access to information (27–30) which could come from extension, media and the farmers' social network (28, 31, 32). This knowledge influences farmers' evaluative capacity (30) which in turn influences farmers' views about the practices (perceptions) (33). Brokensha et al. (34) noted that farmers' perception, knowledge and practice influence how farming decisions are made. It follows therefore that if farmers have positive perception about sustainable production practices, they are likely to practice them. Their perception is however influenced by their knowledge of such practices. On the other hand, the socio-economic characteristics of a farmer determines whether or not he/she will adopt those practices. Carpenter et al. (35) and Prokopy et al. (30) identified farm and farmer characteristics as important factors enhancing a farmer's ability to adopt an innovation and considered it as a resilience capacity. Prokopy et al. (30), Baumgart-Getz et al. (27), and Li et al. (26) noted that important socio-economic variables influencing farmers' adoption decisions (including pro-environmental decisions) are: age, education (formal education and farmer training (extension), marital status, income, farming experience, tenure, social network, labor, place of residence, capital, information and so on. However, a short coming of this model is that is does not take care of other exogenous factors which could influence an individual's choice of using or not using a particular practice. This was taken care of in the study by adapting and modifying the TPB.

## Materials and Methods

The study was conducted in Enugu State, Nigeria. The State is located in the Southeast Geo-political Zone of the Country, lying between latitude 5° 56'N and 7°06'N, and longitude 6°53'E and 7°55'E (36). Multistage sampling procedure was used to select ninety six (96) ruminant farmers that were used for the study. At the first stage, two Agricultural zones (Nsukka and Awgu) were selected through purposive sampling technique from the six agricultural zones in the State. At the second stage, two blocks were selected from each zone using simple random technique giving a total of four blocks. The blocks were Nsukka and Igbo-Etiti (Nsukka zone), Awgu and Aninri (Awgu zone). At the third stage, two circles were selected from each block through simple random sampling technique to give a total of eight circles. The circles were Eziani and Obukpa from Nsukka Block, Ekwegbe, and Ozalla from Igbo-Etiti Block, Mgbowo, and Akwu from Awgu Block, Amorji, and Amokwe from Aninri Block. At stage four, from a list of ruminant farmers provided by the extension worker for each circle, 12 farmers each were selected through systematic random sampling technique. Thus, the total sample size for the study was ninety-six (96).

Semi-structured interview schedule with open-ended sections was used in data collection. To ensure face validity, the interview schedule was validated by three experts while a pre-test was done to ensure reliability of instrument. Written consent was presented and read out to the respondents.

(37) noted two ways of measuring sustainability indicators as: (a) practice-based indicators or action-oriented indicators, i.e., using information on farmers' practices or other causal variables (corresponding to most of pressure indicators), and (b) effect-based indicators or result-oriented indicators, i.e., based on an assessment of the effect at different stages of the cause-effect chain (from emission to impact indicators). For the purpose of this paper, sustainability was measured with practice-based indicators. The study used farmers' practices as a measure of their use of sustainable production practices in ruminant production. Respondents indicated sustainable production practices (SPP) they used for climate change adaptation and mitigation. Each adaptation and mitigation practice used was scored one and a sum of the scores was generated for each respondent. Socio-economic variable were measured as sex (Male = 1, Female = 2), age (years), educational level (years), household size (number of individual in a household), access to extension (number of times in the past 1 year), access to veterinary service (number of times in past 1 year), years of farming experience (number of years), years of ruminant farming experience (number of years), access to credit facilities (Yes = 1, No = 0), annual household income (in naira), annual income form ruminant production (naira). Challenges encountered using sustainable practices were stated and rated on a four-point Likert-type scale of great extent (4), moderate extent (3), little extent (2), and No extent (1) with a mean/cut-off point of 2.5. Any variable with mean of 2.5 and above is accepted as a challenge faced by ruminant farmers in the use of SPP. All interview and discussions were done using local dialect. Data were analyzed using percentage and mean scores, and multiple regression. The socioeconomic factors influencing use of sustainable production practices were measured with a regression model as presented in the model below.


$$\begin{array}{l} \text{Y}=\alpha +\beta_{1}{\text{X}}_{1}+\beta_{2}{\text{X}}_{2}+\beta_{3}{\text{X}}_{3}+\beta_{4}{\text{X}}_{4}+\beta_{5}{\text{X}}_{5}+\beta_{6}{\text{X}}_{6}+\beta_{7}{\text{X}}_{7}\\ \;+\beta_{8}{\text{X}}_{8\ldots \ldots \ldots }+\beta_{12}{\text{X}}_{1}+U \end{array}$$

Where:

Y = Use of sustainable production practice (Number of sustainable production practices used by the ruminant farmer)

β_1−_ β_15_ = Regression coefficient

X_1_= Age (years)

X_2_ = Sex (Male = 1, Female = 0)

X_3_ = Marital status [Married = 1 (Living with spouse), Not married (Not living with spouse) = 0]

X_4_ = Educational level [Educated=1 (any form of formal education), not educated =0 (no formal education]

X_5_= Years of farming experience (years)

X_6_= Years of experience in ruminant production (years)

X_7_= Household size (number of people living under the same roof and having at least one meal per day together)

X_8_= Extension contact in the last 1 year (Yes = 1, otherwise = 0)

X_9_= Access to veterinary services in the last 1 year (Yes = 1, otherwise = 0)

X_10_= Access to credit facilities in the last 1 year (Yes = 1, otherwise = 0)

X_11_= Monthly household income (naira)

X_12_= Annual income from ruminant production (naira)

U = Error team

Pearson moment correlation was used to determine relationship between knowledge level of ruminant farmers on climate change (KLRFCC) and their use of sustainable production practices (SPP). Knowledge statements were generated for each of causes, effects, and adaptation and mitigation measures to climate change. Respondents reacted to a set of thirty (34) KLRFCC test statements by indicating “True” or “False”. Each correct answer was scored “1” while an incorrect answer was scored “0”. A composite score was therefore generated for each respondent for knowledge on climate change and actual use of sustainable production practices (SPP). The relationship was measured at 0.05 probability level.

## Results

### Climate Risks Faced by Ruminant Farmers

A high proportion of the respondents (88.5%) experienced reduced feed/pasture availability; and 94.8% indicated that climate change has resulted in increased price of grains and feed supplement as shown in Table 1. As uncertainties in onset and duration of rains increase as a result of climate change, the quantity and quality of pastures decline and farmers would resort to feed supplementation in order to cope. The competition for feed supplements will no doubt lead to high prices which means more financial pressure on the ruminant farmers. Also, 89.6% perceived increased livestock disease occurrence while 88.5% experienced increased mortality of animals due climate change. Higher risks of infection as a result of high temperatures could overwhelm the coping capacity of ruminant farmers leading to increased mortality of the animals. Similarly, 84.4% of the respondents indicated reduced growth rate and 81.3% perceived lower feed intake. Reduced growth rate is directly linked to low feed intake. When feed intake of animals are affected, major metabolic processes are retarded leading to poor growth rate, reduced milk production, low resistance to diseases and death may result. About 60.4% experienced heat stress on their animals and 54.2% experienced reduced water availability. High temperatures causes drought and heat stress on animals. These triggers physiological disorders and reduced activity in ruminants. This corroborates Malami and Tukur (38) that climate change has led to reduction on feed resources, loss in weight, increased mortality of young animals, increased heat load on the animals from cloudless skies for most part of the year, increased diseases and pest incidence in ruminant production. These effects will no doubt result to low production thereby impacting negatively on the farmers' income. Climate change is therefore hampering sustainable livestock production with the result that availability, accessibility and affordability of animal protein will be greatly affected.

**Table 1:** Climate risks faced by ruminant farmers.

**Risks factors**	**Frequency**	**Percentage**
Heat stress on animals	58	60.4
Lower feed intake	78	81.3
Reduced growth rate	81	84.4
Reduced milk production	7	7.3
Reduced milk quality	4	4.2
Reduced feed/pasture availability	85	88.5
Reduced water availability	52	54.2
Reduced quality of pasture available	44	45.8
Increase livestock diseases occurrence	86	89.6
Increased mortality of animals	85	88.5
Reduced meat quality	4	4.2
Reduced fertility	27	28.1
Increase price of grain/feed supplement	91	94.8
Change in the distribution of pests	10	10.4
Cold stress	9	9.4

### Sustainable Production Practices of Farmers for Climate Change Adaptation and Mitigation

Results of the various adaptation options used by ruminant animal as contained in Table 2 show that almost all (99.0%) of the respondents adapted to the effects of climate change by diversification with non-farming businesses. Ruminant farmers will better adapt to the often sudden and devastating effects of climate change like flooding by engaging in other income generating activities that are not highly dependent on weather events. Most (96.9%) of the respondents adapted by providing sunshade, and 95.8% adapted by ensuring adequate ventilation in the pens. Provision of sunshade and adequate ventilation of pens basically used as measures to reduce the effects of high temperatures which could predispose animals to heat stress and certain ill-health conditions. Most (94.8%) of the respondents also adapted by use of local breeds. Some local breeds are more resistant to extreme weather and have developed immunity to diseases prevalent in the local environment. The most common local breed present in the study area is the West African Dwarf (WAD) goat and sheep which are well-known to be resistant to trypanosomiasis prevalent in humid parts of the country. Also, 92.7% adapted by diversifying livestock production with crop farming. Diversification of farming enterprise helps farmers to withstand the potential economic losses associated with climate shocks. It increases the resilience of livelihoods to climate impacts (39).

**Table 2:** Sustainable production practices used by ruminant farmers for climate change adaptation and mitigation.

**Adaptation options**	**Percentage scores**	**Mitigation options**	**Percentage scores**
Reducing stocking density	**79.2**	Planting of trees around animal houses	22.9
Provision of sun shade	**96.9**	Reduced manure storage time	4.2
Adequate ventilation of pens	**95.8**	Providing bedding materials during cold	7.3
Use of resistant breeds	**84.4**		
Medication/treatment of animals	**63.5**	Using supplementary feeding	**83.3**
Diversification with non-farming businesses	**99.0**	Frequent removal of effluents	**94.8**
Diversification of livestock	**88.5**	Using rotational grazing system	24.0
Saving of animal feed (hay, straw, silage, etc.)	3.1	Intensive rearing of animals/home feeding	**87.5**
Feeding with higher proportion of concentrates	6.3	Provision of vegetative cover (grasses) around animal farm to reduce heat radiation from the soil	4.2
Provision of plenty fresh drinking water	**89.6**	Reduce temperature in manure storage	3.1
Use of local breeds resistant to prevailing climate conditions	**94.8**	Addition of essential oils to animal diet reduce emissions	4.2
Vaccination of animals	4.2	Diversification of animal feed	**90.6**
Diversification with crop farming	**92.7**	Reducing stocking density	**79.2**
Harvesting forage for ensiling at an early stage of maturity	3.1	–	–
Seasonal migration (movement) of animals	3.1	–	–
Cross breeding with resistant breeds	12.5	–	–

*Bold values: Sustainable production practices used*.

Similarly, the majority (89.6%) of the respondents adapted by providing plenty drinking water for animals, 88.5% adapted by diversifying their livestock types, while 87.5% engaged in intensive rearing/home feeding of animals. Provision of plenty fresh drinking water can help cushion the effect of heat stress while diversification of livestock can help reduce the infestation and spread of weather- related illnesses that are more prevalent in a particular specie of animal (10). Also, 63.5% adapted by medication/treatment of animals. Regular treatment of diseases improves herd health and productivity thereby reducing death of animals and consequent economic loss resulting from climate change.

These results show that ruminant farmers are adapting to climate change by using various sustainable management practices. Improved management practices which do not increase harm done to the environment are advocated for sustainable ruminant production (40). These practice ensure that farmers continue to increase production to enhance profitability of their livelihood activities while constituting minimal or no harm to the environment.

Results show that respondents adopted mitigation options as indicated by 94.8% who engage in frequent removal of effluents, while 90.6% diversify animal feed and 83.3% used supplementary feeding. Deficiencies and metabolic diseases caused by feed scarcity and poor quality feed can be cushioned through feed diversification and use of supplements while removal of effluents can ameliorates the build-up of GHGs. About 87.5% engage in intensive rearing and 79.2% engage in reduction of stocking density which leads to lesser emissions of GHGs (41).

It is noteworthy however that a number of sustainable practices were yet to be embraced by a good number of ruminant farmers. A closer look at the results suggests that respondents were less engaged in mitigation practices. Planting trees around animal houses contributes to carbon sequestration as well as providing shading to animal houses in extreme weather conditions (10, 16), feeding with higher proportion of concentrates reduces methane release during enteric fermentation (42), addition of essential oils in feed reduces methane release during enteric fermentation (43, 44), reduced manure storage time lowers the emissions of nitrous oxide and methane by volatilization (10, 45), while reduced temperature in manure storage (manure cooling) reduces methane formation (46). The implication of the low use of the mitigation measures is that more harm will continue to be done on the environment by ruminant farmers in the area. On the other hand, the results reveal that proactive measures to possible effects of impending climate catastrophes were not widely practiced by the respondents. For instance, harvesting silage at early stage of maturity helps to ensure nutritious herbage (16, 42) even during drought. seasonal migration of animals helps farmers avoid impending climate risks like drought, flood, and disease epidemics (47), storing of animal feed can help ensure availability of enough quantity of feed in periods of scarcity such as drought, while immunization/vaccination of animals helps to prevent disease epidemic among herds which can be triggered by climate extremes (39). Harvesting of silage and storing of forage for use during drought periods could reduce migration and the consequent incessant farmer-herder conflicts which has resulted to unprecedented loss of lives and properties in the country.

### Socio-Economic Factors Influencing Respondents' Use of Sustainable Practices

Table 3 shows the influence of socio-economic characteristics of respondents on their use of sustainable production practices. The overall result was significant (*F* = 2.829, *p* = 0.004), implying that socio-economic characteristics of ruminant farmers had significant influence on their use of sustainable production practices. The regression results show that among the socio-economic factors, access to veterinary services (*t* = 2.056, *p* < 0.05) had significant positive influence on use of sustainable production practices. This implies that ruminant farmers with access to veterinary services engaged more sustainable production practices than those without access. This could be attributed to the fact that veterinarians supply farmers with relevant advice on sustainable strategies used in dealing with the impacts of climate change on health of ruminant animals. Also, monthly household income had significant positive influence (*t* = 3.582, *p* < 0.05) on the use of sustainable production practices. Household income in this study refers to the totality of income generated (estimated monthly) by all members of a household including farm and non-farm incomes. This means that higher income status of households enhances the use of sustainable production practices. The result may stem from the fact that a number of sustainable production practice may require extra expenses and household members can willingly and easily offer financial assistance to the household member rearing ruminants. The implication of this is that relevant bodies such as development agencies, governments and financial institutions could support the use of sustainable production practices through programmes aimed at increasing the financial base of ruminant farmers. Terfa and William (48) stated that since rural farm households in sub-Saharan Africa are vulnerable to climate change as they have low financial base, it is crucial to examine how financial inclusion can enhance their resilience to the increasing impact of climate change.

**Table 3:** Socio-economic factors influencing respondents' use of sustainable practices.

**Model**	**Unstandardized coefficients**		**Standardized coefficients**		
	**B**	**Std error**	**Beta**	**T**	**Sig**
Constant	9.911	1.349		7.346	0.000
Age	0.046	0.031	0.309	1.493	0.141
Sex	−0.460	0.499	−0.122	−0.922	0.360
Marital status	−0.171	0.613	−0.033	−0.280	0.781
Educational level	0.758	0.584	0.173	1.298	0.199
Years of farming experience	−0.010	0.035	−0.069	−0.297	0.768
Years of experience in ruminant production	−0.015	0.030	−0.087	−0.502	0.618
Size of household	0.021	0.107	0.022	0.193	0.847
Extension contact	−3.586	1.966	−0.195	−1.823	0.073
Access to veterinary services	0.901	0.438	0.239	2.056	**0.044**
Access to credits facilities	0.367	0.949	0.044	0.387	0.700
Estimated monthly income	5.927-5	0.000	0.473	3.582	**0.001**
Annual income from ruminant production	−8.345-5	0.000	−0.382	−2.635	**0.011**

*Dependent variable: number of sustainable production practices, P = 0.05, R = 0.604, R^2^ = 0.365, Adjusted R^2^ = 0.236. Bold values: Significant values*.

On the other hand, results show that annual income from ruminant production had significant negative influence (*t* = −2.635, *p* < 0.05) on use of sustainable practices. This could mean that larger farms tend to engage in unsustainable practices than smaller farmers. According to Lin et al. (49), large scale livestock production increases the impact of livestock on GHGs emissions which includes a variety of production-related activities such as over grazing, enteric fermentation, feed-crop production with fertilizers and burning of fossil fuel through transportation of inputs, outputs and products. This result implies that ruminant farmers tend to overlook their production activities once their herd size gets larger. This is attributable to poor management arising from the financial, labor and other demands required in taking care of large herd size. Advocates of sustainable development could target larger ruminant farms in the provision of incentives for enhancing sustainable production of ruminant.

The R square value is the proportion of the variability in the use of sustainable production practices which was explained by the regression model. The adjusted R square is the estimate of *r*^2^ for the population. Therefore, access to veterinary services, monthly income and income from ruminant production were able to explain 24% of the variance in the use of sustainable practices by respondents. The TPB posits that individuals will adopt a behavior which they think they can benefit from and which they have capacity to use within their own specific circumstances. This result support the theory in that it shows that the ruminant farmers can use those sustainable practices for which they have financial capacity to adopt.

### Relationship Between Respondents' Knowledge Level on Climate Change (KLCC) and Their Use of Sustainable Production Practices (SPP)

Table 4 shows a Pearson Moment correlation between ruminant farmers' knowledge level on climate change (KLCC) and their use of sustainable production practices (SPP). Results show that there is a significant positive correlation (*r* = 0.426, *p* < 0.05) between KLCC and their use of SPP. This means that higher knowledge level of ruminant farmers on climate change enhances use of sustainable production practices. Nwobodo and Agwu (18) had noted that climate change knowledge level of farmers needed to be improved in order to foster effective adaptation and mitigation as continuous neglect of cognitive adaptive capacity of individual actors on climate change will undermine efforts of attaining the goals of current and future adaptation strategies. The implication of this is that climate change communicative interventions such as trainings and symposiums should target ruminant farmers to boost their knowledge and enhance their use of sustainable practices. Such interventions could focus more on activities that enhance mitigation by reducing greenhouse gas emissions thereby reducing catastrophic events resulting from climate change. This will promote long term resilience of ruminant farming systems. Pretty and Bharucha (50) suggest improvement of farmers' knowledge and capacity through the use of farmer field schools, videos and modern information communication technologies.

**Table 4:** Correlation between knowledge level and number of sustainable practices used.

		**Knowledge level**	**Number of sustainable production practices used**
Knowledge level and use of SPP	Correlation coefficient	1	0.426
	Sig. (2-tailed)		0.000
	N	96	96

*Source: Field data, 2018–2019*.

### Challenges to Use of Sustainable Production Practices

The challenges (Table 5) farmers encounter in using sustainable practice in ruminant production were: lack of funds (*M* = 3.71, SD = 0.78), land scarcity (*M* = 2.52, SD = 1.24), high prevalence of animal diseases (*M* = 2.77, SD = 0.83), and high cost of drugs (*M* = 2.68, SD = 1.11). Land scarcity and lack of funds hinders expansion of ruminant production enterprise. Farmers are limited in the number of animals they can keep because of insufficient fund and limited land area. Farmers in the area could only construct small pens within the available land which can only contain limited number of animals. This will restrict the spaces available and could subject the animal to overcrowding. Respondents narrated how livestock diseases such as tryponosomiasis, mastitis, brucellosis, foot and mouth disease, ecto- parasites and endo-parasites are reported had constrained their ruminant production enterprise. They stated that high cost of drugs and veterinary services to attend to sick animals is very high leaving many of the farmers without regular access veterinary services. Offor et al. (51) had noted that insufficient fund and disease incidence were the major constraints identified by small ruminant farmers. The increased incidences of diseases outbreak resulting from climate change without regular access to veterinary services leads to high mortality which translates to huge economic losses to ruminant farmers.

**Table 5:** Challenges to use of sustainable production practices in ruminant production.

**Constraints**	**Mean**	**Std deviation**
Lack of funds	**3.71**	0.78
High cost of feeds	2.41	1.25
High prevalence of animal diseases	**2.77**	0.83
Poor educational level of farmers	2.00	1.01
Failed government policies	1.70	0.95
Urbanization	1.14	0.57
Inadequate storage facilities	1.51	0.81
Glut when marketing during shock	1.27	0.62
Inadequate extension services	2.37	1.08
Inadequate manpower	2.20	1.15
Poor awareness on sustainable production practices	2.22	1.16
Water scarcity	1.70	0.81
Pressure on grazing lands	1.45	0.80
Lack of access to improved breeds	1.56	0.90
Theft	1.47	0.94
Inadequate modern farm input	1.56	0.89
Lack of good management skills	1.99	1.05
High cost of drugs	**2.68**	1.11
Transportation issues	1.39	0.64
Inadequate basic infrastructure	1.50	0.82
Land scarcity	2.52	1.24
Poor attitude to animal production	1.71	1.10
Cultural influence	1.03	0.18

*Cut-off = 2.5. Bold values: equal to or greater than the cut-off point*.

The standard deviations from almost all the results were less than zero (0), except for high cost of drugs. This suggests that respondents had convergent view on the challenges they faced in using sustainable production practices. However, respondents had little bit of divergent view on high cost of drug. This could mean that high cost of drug is relative to the income level of each of the respondents. Most of the ruminant farmers were poor and would find it costly to afford basic drugs for their animals. Having identified diseases outbreak as a major constraint, the farmers would not have complained about the cost of drugs if they were high income earners.

## Discussion

Ruminant farmers make use of sustainable production practices. However, they made more use of adaptation practices and little of mitigation practices. They may have engaged more in adaptation because those measures are more or less *ad hoc*, offering immediate relief to the effects of climate change. This is attributable to their low scale of production which does not encourage long term investments especially in practices that do not yield immediate results. Also, farmers may not be inclined to adopting mitigation measures if they do not see a tangible link between such measures and farm productivity and/or household food security. Wollenberg et al. (52) argue that smallholder farmers in developing countries prioritize immediate benefits and are more likely to adopt mitigation measures if they perceive co-benefits or outcomes in using such measure in enhancing productivity and improved household food security. Ruminant farmers could be encouraged to adopt mitigation strategies through schemes such as payment for ecosystem services, increased prices for sustainable low carbon impact products. Agricultural development agencies and governments could mainstream mitigation of climate change through such policies that offer incentives to ruminant farmers who adopt mitigation measures. Although the number of sustainable productive practices used appears to be limited in terms of explaining the use of SPP. However, the challenges to use throws explained other factors that could constrain or enhance use. Lack of funds, high prevalence of animal diseases, high cost of drugs and land scarcity were the challenges faced by farmers in using sustainable production practices. Having access to veterinary services, estimated monthly income were the socio-economic factors that positively influence farmers use of sustainable practices while annual income from ruminant production negatively influence farmers use of sustainable practices.

## Conclusion

Relevant policies that promote financial inclusion such as improved access to credits, loans and grants will help ruminant farmers build resilience while minimizing their contributions to climate change. The significant positive correlation between knowledge level on climate change and use of SPP implies that more knowledge of climate change issues could translate to better adaptation to and mitigation of climate change. Therefore, **a**gricultural extension agencies should prioritize adaptation and mitigation in its tool kit. Famer-to-farmer extension should be encouraged in order to offer ruminant farmers more opportunities to learn from fellow farmers who could act as climate change vanguards by development agencies. More sophisticated adaptation and mitigation strategies could be introduced to farmers in the area. Governments could make provisions for veterinary services at a subsidized rate for farmers.

## Data Availability Statement

The original contributions presented in the study are included in the article/supplementary material, further inquiries can be directed to the corresponding author/s.

## Ethics Statement

The study involving human participants was reviewed and approved by the Department of Agricultural Extension, University of Nigeria, Nsukka. Written informed consent of the participants was received during the field survey.

## Author Contributions

CN conceived and conceptualized the study and wrote the draft of the manuscript. BN wrote the background, collected the data, and wrote part of the manuscript. JI scrutinized and improved the manuscript. VO and RO read and corrected the background and methodology. All authors contributed to the article and approved the submitted version.

## Conflict of Interest

The authors declare that the research was conducted in the absence of any commercial or financial relationships that could be construed as a potential conflict of interest.

## Publisher's Note

All claims expressed in this article are solely those of the authors and do not necessarily represent those of their affiliated organizations, or those of the publisher, the editors and the reviewers. Any product that may be evaluated in this article, or claim that may be made by its manufacturer, is not guaranteed or endorsed by the publisher.

## References

[1] Rivera-Ferre MG Lopez -i- Gelats F Howden M Smith P Morton JF Herrero M. Re-framing the climate change debate in the livestock sector: mitigation and adaptation options. WIREs Clim Change. (2016) 7:869–892. 10.1002/wcc.42125855820

[2] Food and Agriculture Organization of the United Nations. Greenhouse Gas Emissions from Ruminant Supply Chains: A Global Life Cycle Assessment. Rome: FAO (2013).

[3] Humane Society International. An HSI Report: The Impact of Animal Agriculture on Global Warming and Climate Change (HSI) 2011. Retrieved from: http://www.humanesociety.org/assets/pdfs/farm/hsus-the-impact-of-animal-agriculture-on-global-warming-and-climate-change.pdf

[4] Varijakshapanicker P Mckune S Miller L Hendrickx S Balehegn M Dahl GE. Sustainable livestock systems to improve human health, nutrition, economic status. Anim Front. (2019) 9:39–50. 10.1093/af/vfz041 32002273 PMC6951866

[5] Zhang YW McCarl BA Jones JPH. An overview of mitigation and adaptation needs and strategies for the livestock sector. Climate. (2017) 5:95. 10.3390/cli5040095

[6] Tessema W Ingenbleek P Trijp H. Pastoralism sustainability and marketing: a review. Agron Sustain Dev. (2014) 34:75–92. 10.1007/s13593-013-0167-425687702

[7] Behnke GD Pittelkow CM Nafziger ED Villamil MB. Exploring the relationship between Greenhouse emissions, yield, and soil properties in cropping systems. Agriculture. (2018) 8:62. 10.3390/agriculture8050062

[8] Orheruata AM Omoyakhi JM. Livestock-environment interaction: issues and options in Nigeria. J Appl Sci Environ Manage. (2008) 12:129–33. 10.4314/jasem.v11i4.55178

[9] Sejian V. Climate change: impact on production and reproduction, adaptation mechanism and mitigation strategies in small ruminants. A review. Indian J Small Ruminants. (2013) 19:1–21. 10.1163/15707563-00002438

[10] Rojas-Downing MM Pouyan AN Harrigan T Woznicki SA. Climate change and livestock: impacts, adaptation, and mitigation. Clim Risk Manage. (2017) 16:145–63. 10.1016/j.crm.2017.02.001

[11] Biobaku KT Amid SA. Predisposing factors associated with diseases in animals in Nigeria and possible botanical immunostimulants and immunomodulators: a review. Bangl J Vet Med. (2018) 16:87–101. 10.3329/bjvm.v16i1.37381

[12] Shinde AK Sejian V. Sheep husbandry under changing climate scenario in India: an overview. Indian J Anim Sci. (2013) 83:998–1008.

[13] Food and Agriculture Organization of the United Nations. Desertification. Available online at: http://www.fao.org/desertification/default.asp?lang=en, on(accessed April 18, 2019)

[14] Ashraf A Mohammad MD Basharat MW Showkat AS Mir S Majid S. Climate change and infectious diseases of animals: a review. J Entomol Zool Stud. (2017) 5:1470–7.

[15] Anyanwu NJ Ogualu JO Odoemena VU Kalio GA Epke II. Sheep and goat farming in Imo State Southeast Nigeria: A traditional vocation at the verge of extinction. Nigerian J Anim Product. (2020) 47:237–46. 10.51791/njap.v47i4.70

[16] Hoving IE Stienezen MWJ Hiemstra SJ van Dooren HJ Buisonje FE. Adaptation of Livestock Systems to Climate Change, Funstions of grassland, Breeding, Health and Housing. Wageningen UR Livestock Research (2014).

[17] Velten S Leventon J Jager N Newig J. What is sustainable agriculture? A systematic review. Sustainability. (2015) 7:7833–65. 10.3390/su7067833

[18] Nwobodo CE Agwu AE. Knowledge level of youth farmers on climate change in Benue State of Nigeria. Afr J Agric Res. (2015) 10:2875–81. 10.5897/AJAR2015.9680

[19] Giuseppe P Ana HDF Bruno S Agostino S Luigi C Nicola L. Sustainable ruminant production to help feed the planet. Italian J Anim Sci. (2017) 16:140–71. 10.1080/1828051X.2016.126050032585906

[20] Henry B Charmley E Eckard R Gaughan JB Hegarty R. Livestock production in a changing climate: adaptation and mitigation research in Australia. Crop Pasture Sci. (2012) 63:191–202. 10.1071/CP1116928948418

[21] Si H Shi J Tang D Wen S Miao W Duan K. Application of theory of planned behavior in enviromnetal scienc: a comprehensive bibiometric Analysis. Int J Environ Res Public Health. (2019) 16:1–26. 10.3390/ijerph16152788 31382712 PMC6695987

[22] Fishbein M Ajzen I. Belief, Attitude, Intention, Behaviour: An Introduction to Theory Research. London: Addison-Wesley. (1975).

[23] LaMorte WW. The theory of Planned Behavior. (2019). Available online at: http://sphweb.bumc.bu.edu/otlt/MPHModules/SB/BehavioralChangeTheories/BehavioralChangeTheories3.html on 05/01/2020

[24] Ryan S Carr A. Applying the biopsychological model to the management of rheumatic disease. In: Conner M, editor. Rheumatology. International Encyclopedia of the Social and Behavioural Sciences (Amsterdam: Elsevier) (2010).

[25] Ajzen I Joyce N Sheikh S Cote NG. Knowledge and the prediction of behaviour: the role of information accuracy in the theory of planned behavior. Basic Appl Soc Psychol. (2011) 33:101–17. 10.1080/01973533.2011.568834

[26] Li D Zhao L Ma S Shao S Zhang L. What influences an individual's pro-environmental behavior? A literature review. Resour Conserv Recycl. (2019) 146:28–34. 10.1016/j.resconrec.2019.03.024

[27] Baumgart-Getz A Prokopy LS Floress K. Why farmers adopt best management practice in the United States: a meta-analysis of the adoption literature. J Environ Manage. (2012) 96:17–25. 10.1016/j.jenvman.2011.10.006 22208394

[28] Greiner R Patterson L Miller O. Motivations, risk perceptions and adoption of conservation practices by farmers. Agric Syst. (2009) 99:86–104. 10.1016/j.agsy.2008.10.003

[29] Lambrecht I Vanlauwe B Merckx R Maertens M. Understanding the process of agricultural technology adoption: mineral fertilizer in eastern DR Congo. World Dev. (2014) 59:132–46. 10.1016/j.worlddev.2014.01.024

[30] Prokopy L Floress K Klotthor-Weinkauf D Baumgart-Getz A. Determinants of agricultural best management practice adoption: evidence from the literature. J Soil Water Conserv. (2008) 63:300–11. 10.2489/jswc.63.5.300

[31] Knowler D Bradshaw B. Farmers' adoption of conservation agriculture: a review and synthesis of recent research. Food Policy. (2007) 32:25–48. 10.1016/j.foodpol.2006.01.003

[32] Pannell DJ Marshall GR Barr N Curtis A Vanclay F Wilkinson R. Understanding and promoting adoption of conservation practices by rural landholders. Austral J Exp Agric. (2006) 46:1407–24. 10.1071/EA0503728948418

[33] Bwambale N. Farmers' knowledge, perceptions, and socioeconomic factors influencing decision making for integrated soil fertility management practices in Masaka and Rakai Districts, Central Uganda. Graduate Theses and dissertation. Iowa State University, Ames, IA, United States (2015).

[34] Brokensha DW Warren DM Werner O. Indigenous Knowledge Systems and Development. Maryland: University Press of America (1980).

[35] Carpenter S Walker B Anderies JM Abel N. From metaphor to measurement: resilience of what to what? Ecosystems. (2001) 4:765–81. 10.1007/s10021-001-0045-9

[36] Ezike JO. Delineation of old and new Enugu State. Bulletin of the Ministry of Works, Lands and Survey, Enugu State. Government Bulletin, Enugu, Ministry of Works (1998).

[37] Latruffe L Diazabakana A Bockstaller C Desjeux Y Finn J Kelly E. Measurement of sustainability in agriculture: a review of indicators. Stud Agric Econ. (2016). 118:123–130. 10.7896/j.1624

[38] Malami BS Tukur HM. Effects of climate change on livestock production in semi-arid Nigeria: pastoralists' perception and coping strategies. Usmanu Danfodiyo University Sokoto-Nigeria. (2017) 1:16–23.

[39] Delia G Bett B Lindahl J Robinson T. Climate and Livestock Disease: Assessing the Vulnerability of Agricultural Systems to Livestock Pests Under Climate Scenarios. Montpellier: CGIAR. (2015).

[40] Ifeanyi-Obi CC Nnadi FN. Climate change adaptation measures used by farmers in Southsouth Nigeria. J Environ Sci Toxicol Food Technol. (2014) 8:1–6. 10.9790/2402-08410106

[41] International Fund for Agricultural Development. Livestock and Climate Change. Livestock Thematic Papers (IFAD), (2009). Available online at: enterprise-development.org/wp-content/uploads/Livestock_and_Climate_Change.pdf (accessed January 8, 2020).

[42] Haque MN. Dietary manipulation: a sustainable way to mitigate methane emissions from ruminants. J Anim Sci Technol. (2018) 60:1–10. 10.1186/s40781-018-0175-7 29946475 PMC6004689

[43] Yatoo MA Chaudhary LC Agarwal N Chaturvedi VB Kamra DN. Effect of feeding of blend of essential oils on methane production, growth, and nutrient utilization in growing buffaloes. Asian Australas J Anim Sci. (2018) 31:672–6. 10.5713/ajas.16.0508 28231698 PMC5930277

[44] Benchaar C Greathead HMR. Esential oils and opportunities to mitigate enteric methane emissions from ruminants. Fuel Energy Abstracts. (2011) 166:338–55. 10.1016/j.anifeedsci.2011.04.024

[45] Jokela B Laboski C Andraski T. Manure Application Methods and Timing Effects on Emission of Ammonia and Nitrous Oxide. (2019). Accessed online at: https://lpelc.org/manure-application-method-and-timing-effects-on-emission-of-ammonia-and-nitrous-oxide/

[46] Webb J Sorensen P Velthof G Amon B Pinto M Rodhe L. Assessment of the variation of Manure Nitrogen Efficiency throughout Europe and an appraisal of means to increase manure-N efficiency. Adv Agron. (2011) 119:371–442. 10.1016/B978-0-12-407247-3.00007-X

[47] Berhe M Hoag D Tesfay G Tadesse T Oniki S Kagatsume M. The effects of adaptation to climate change on income of households in rural Ethiopia. Pastoral Res Policy Pract. (2017) 7:1–15. 10.1186/s13570-017-0084-233824703

[48] Terfa WA William MF. Climate change and financing adaptation by farmers in northern Nigeria. Fin Innovat. (2018) 4:1–17. 10.1186/s40854-018-0094-0

[49] Lin BB Chappell MJ Vandermeer J Smith G Quintero E Berner-Kerr R. Effects of industrial agriculture on climate change and the mitigation potential of small-scale agro-ecological farms. CAB Rev. (2011) 6:1–18. 10.1079/PAVSNNR20116020

[50] Pretty J Bharucha ZP. Sustainable intensification in agricultural systems. Ann Bot. 114:1571–1596. (2014). 10.1093/aob/mcu205 25351192 PMC4649696

[51] Offor EI Ekweanya NM Oleka AC. Effects of socio-economic factors on small ruminant production in Ohafia Agricultural Zone of Abia State. Agro Sci J Trop Agric Food Environ Extens. (2018) 17:7–11. 10.4314/as.v17i3.2

[52] Wollenberg E Higman S Seeberg-Elverfeldt C Neely C Tapio-Biström ML Neufeldt H. Helping Smallholder Farmers Mitigate Climate Change. CGIAR CCAFS (2012). Available online at: https://cgspace.cgiar.org/bitstream/handle/10568/21730/CCAFS_Brief05_Finalamended(web)_LR.pdf?sequence=1

